# Effects of Drought and Media-Reported Violence on Cattle Fever Tick Incursions

**DOI:** 10.3389/fvets.2020.00373

**Published:** 2020-07-03

**Authors:** Jada M. Thompson, Amy H. Delgado, Hallie S. Hasel, Denise L. Bonilla

**Affiliations:** ^1^Department of Agricultural and Resource Economics, University of Tennessee, Knoxville, Knoxville, TN, United States; ^2^U.S. Department of Agriculture, Animal and Plant Health Inspection Service, Veterinary Services, Fort Collins, CO, United States

**Keywords:** cattle fever ticks, tick control, environmental conditions, drought, media-reported violence, border violence

## Abstract

Ectoparasites, such as cattle fever ticks, and the diseases they carry pose a risk to the global cattle population in reduced productivity and in livability. Tick infestations carry significant economic implications through losses in productivity, increased morbidity, and control costs. Cattle fever ticks were eradicated from the United States through concentrated efforts across state and federal agencies. The Cattle Fever Tick Eradication Program maintains a permanent quarantine and buffer zone along the Texas-Mexico border to monitor and control reincursions of the tick from Mexico due to movements of wildlife or stray animals. The number of apprehensions of stray livestock and changing infestation rates may be influenced by many factors including increases in violence along the border or environmental effects such as weather pattern changes, river levels, or temperature fluctuations. Using annual records of the number of cattle apprehended and infestation rates, an analysis of the effects of media-reported border violence and environmental conditions can provide a unique understanding of cattle fever tick prevention and the challenges control programs face. Results from this analysis suggest that both media-reported violence and weather changes affect the rate at which infested cattle are apprehended, and these effects differ depending on spatial and temporal factors. With continued land use changes, social unrest in endemic areas, and changing weather patterns, the efforts to control and eradicate cattle fever ticks, both in the United States and globally, is likely to be an ongoing concern.

## Introduction

Cattle fever ticks (*Rhipicephalus microplus* and *Rhipicephalus annulatus*) (CFT) are species of ticks that can carry parasites such as *Babesia bigemina* or *Babesia bovis* which causes the protozoal disease babesiosis, commonly called cattle fever in the United States (US) or tick fever in other countries. Cattle fever leads to anemia, reduced milk production, loss of weight, increased morbidity and even mortality in infected cattle that are left untreated. These ticks pose a threat to the 1.5 billion cattle globally, especially in tropical areas where the host tick densities are the highest. For US cattle, cattle fever was once a significant animal health epidemic, but through concerted efforts by livestock producers as well as federal and state agencies, the tick vector has been successfully eradicated, and a permanent quarantine area has been established to monitor for reincursions from Mexico where the tick is endemic (1–3). The Cattle Fever Tick Eradication Program (CFTEP) continues to support efforts to ensure the health and well-being of U.S. cattle through vigilant surveillance and response to fever tick incursions.

**Figure 1:**
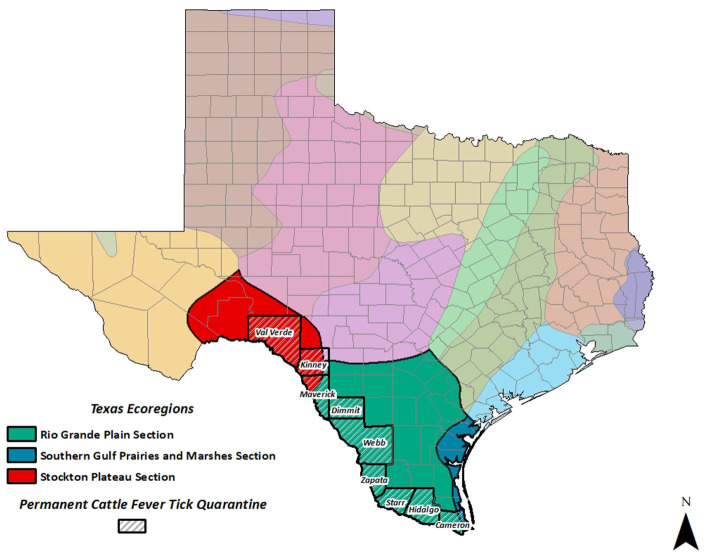
Permanent Cattle Fever Tick Quarantine and Ecoregions of Texas Described by Bailey (6).

According to the Texas Department of Transportation, Texas and Mexico share 1,254 miles of border with 28 international bridges and crossing points, which include a hand-drawn ferry, numerous dams, rail-only, and other crossings (4). The border is defined by the route of the Rio Grande River, the fifth longest river in the US and among the top 20 in the world (About the Rio Grande^1^). The border between the US and Mexico is highly trafficked with over 33 million personal vehicles and 17 million pedestrians crossing Northbound in single year (5). The Permanent Cattle Fever Tick Quarantine zone (PQZ) traverses nearly 580 miles along this border and ranges from 125 yards to nearly 8 miles wide (see Figure 1). The PQZ includes areas of nine south Texas counties: Cameron, Dimmit, Hidalgo, Kinney, Maverick, Starr, Val Verde, Webb, and Zapata. Within the PQZ, livestock producers are required to treat their cattle for ticks, using large dipping vats, spray treatments, or injectable treatments all monitored by USDA and Texas Animal Health Commission (TAHC). Patrolling along the Texas/Mexico border are mounted riders, more familiarly called tick riders, which intercept stray livestock moving across the border. These livestock are checked for ticks, treated, and returned to their owners where possible.

There are many factors that could contribute to changes in the frequency of incursions of cattle fever ticks. Suitable tick environments in terms of host, vegetation, and climate along both sides of the border and extreme weather patterns can cause push and pull effects for ticks as well as for their hosts–cattle and wildlife. Increases in the number of stray animals from Mexico due to economic or regional instability due to violence can lead to increased introductory pressures as farms are abandoned and animals left to fend for themselves. Using data collected from the CFTEP, an analysis of the factors that contribute to cattle fever tick incursions and infestation rates was conducted with a focus on the implications of border violence and environmental effects.

## Background

When first identified, CFT were believed to only infest cattle, but they have been found on a variety of domesticated animals including equids, as well as, wild animals such as white tail deer (*Odocoileus virginianus*), red deer (*Cervus elaphus*), and the invasive nilgai antelope (*Boselaphus tragocamelus*) (5, 7–9). This broad range of free-moving hosts poses unique challenges for surveillance and management of incursions within the PQZ, with changes in host densities possibly leading to increased infestations. Pound et al. (10) showed that increases in densities of white-tailed deer in southern Texas and northern Mexico led to increases in the number of CFT infestations in the US. Eradication of CFT in 14 states of the southeastern US and southern California was achieved by 1943, but total eradication from the US was delayed by the persistence of CFT on deer populations in southern Texas and Florida (8). In addition to mounted patrols, the CFTEP also treats products from deer harvested in the PQZ to ensure CFT are not taken out of the zone. As for controlling CFT, infested cattle are dipped in organophosphates, while deer populations are treated seasonally, February through July, through 1,500–2,000 feeding stations of ivermectin treated corn annually to reduce tick infestations (10–12). However, continued containment of CFT within this high risk region has been challenged by increases in tick resistance to acaricides in Mexico and Texas, movement of sylvatic hosts, such as white tail deer back and forth across the border, and incursions of ticks into U.S. Fish and Wildlife Services Refuges, where treatment or mitigation is extremely difficult, leading to a need for more directed control measures and innovations in control of CFT (5, 13, 14). Alternatives currently in use include resistant cattle selection and exploration of novel chemical and biological controls (15, 16).

While successful, the eradication of CFT in the US has been costly—costs totaling more than $3 billion in today's dollars—and a broad scale reintroduction could be devastating to the US cattle industry in terms of animal health and welfare, leading to costs upward of $1 billion annually (11). This estimated cost of reintroduction includes the cost of tick control (labor, treatments, and opportunity losses of capital associated with control) in addition to the losses in production due to morbidity in beef cattle and milk production losses in dairy cattle, if not leading to widespread mortality, as well as the effects on consumer prices and welfare (17). Co-morbidity would also be a concern, with CFT infestations further exacerbating animal health conditions. Given the severity of the consequences resulting from re-establishment of CFT in the U.S. outside of the PQZ, constant surveillance and effective control in the PQZ is of paramount importance in reducing the risk of reintroduction of CFT to the US.

### Cattle Fever Tick Habitation

While the CFT's hosts may be varied, optimal tick habitats influence the spread and establishment of CFT populations. As a highly adaptable species, CFT are established in tropical and subtropical regions throughout the world (8). In North America, these ticks are endemic only in Mexico, where optimal tick habitat support infestations of 65% of the country with *R. microplus* (13, 18). However, evidence shows that the different CFT species do not have the same optimal habitation. Lohmeyer et al. (19) used data collected on CFT infestations and distributional mapping to provide evidence of a parapatric boundary between the two CFT species. This biogeographical boundary could be a result of environmental factors, genetics, or some combination of the two, but the distinction may provide an indicator of the species of ticks most likely to occur in different areas of the PQZ.

Like many vectors, ticks are susceptible to changes in climate and ecosystems (20). The spread, seasonality, and abundance of CFT are likely affected by climate traits among many other complex factors, and these influences may also have an effect on transmission risk (20). Giles et al. (5) modeled the range expansions of CFT given changes in weather patterns, and predicted increased pressures on the southeast United States, not through cattle movements alone but through changes in optimal tick habitats. The most suitable range for these ticks is currently in Texas and California, but researchers' models suggest expansion of this range into New Mexico and Arizona, with Louisiana, Oklahoma, Arkansas, Mississippi, Alabama, Georgia, and Florida as moderately suitable habitats (5). This expanded range would be consistent with historical tick population in the United States. Changes in weather conditions are likely to influence host population dynamics and the patterns of CFT persistence in the PQZ over time, leading to temporal or spatial shifts in tick incursions and establishment in the PQZ.

### Human Influences on Cattle Fever Ticks

Similar to climate, human directed ecosystem changes can lead to instability and changes in tick habitation and pest pressure. Ecosystem changes include land use, urbanization and urban encroachment of habitats, habitat fragmentation, land divisions, changes in vegetation, along with many other human directed causes (5, 21). In addition to the changes in tick habitats, human directed ecosystem changes can also drive CFT host movements. For example, production intensification of export-destined cattle in the three Mexican states which border the PQZ—Coahuila, Nuevo Leon, and Tamaulipas which make up 33.24% of Mexico's cattle exports—can lead to increased risks for Mexican cattle escaping enclosures or breaking free from a larger herd and crossing the border carrying CFT (13, 22). During drought periods, ranchers may move their animals closer to rivers in search of green forage, which can provide access for river crossings. Additionally, backyard “traspatio” cattle are at risk of becoming lost or stray during distressing periods, such as droughts, financial hardships reducing investment and maintenance of farm infrastructure, or periods of violence leading to farm abandonment. Farm abandonment can be caused by financial, physical, or emotional distress both on producers and the local economies (23). Some criminal activities can lead to farm abandonment through fear and unrest (24). Understanding how human mediated pressures on CFT and tick hosts move into the PQZ can lead to improved planning during these events.

## Materials and Methods

### Data

**Figure 2:**
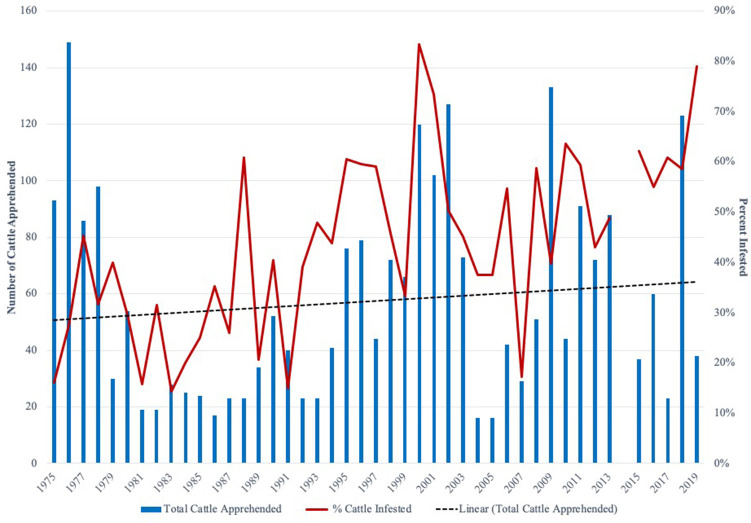
Total cattle apprehended along Texas-Mexico Border and percent of cattle infested with Cattle Fever Ticks from 1975 to 2019*. *2014 excluded from figure as it represents outlier period where 200 cattle which had lived in the Permanent Cattle Fever Tick Quarantine Area for more than a decade were requested to be moved by the Cattle Fever Tick Eradication Program and does not represent cattle apprehended and checked for cattle fever ticks.

The data used in this analysis were collected from various sources. Annual cattle apprehension data were recorded by CFTEP personnel and tick riders from 1975 to 2019^2^ for the nine counties in the PQZ. This data included the total number of cattle apprehended, the number of infested and non-infested cattle, and the county where the cattle were apprehended. Due to limited apprehensions, Kinney and Dimmit counties were dropped from all empirical models. These counties, while technically in the PQZ, have very little of the county abutting the border with Mexico. For the purposes of this analysis, we were interested in the changes in the number of infested cattle (*InfestedCattle*) over time. Overall trends visualized in Figure 2, show an increase in cattle apprehended and an increase in infestation rates over the time period examined. The factors studied in this analysis aim to explain this variability in the apprehensions and infestations over time.

Apprehensions are a function of the quantity and quality of tick riders that scout, track, and apprehend stray cattle throughout the PQZ. In order to capture the fluctuation of available labor, the number (in thousands) of horseback patrol hours (*RiverPatrolHours*) was collected from the CFTEP. The use of tick rider patrol hours accounts for overall patrol counts as well as the various number of tick riders for any given period. Annual patrol hours were only available in aggregate from 1990 to 2019 for the entire PQZ, so county specific hours could not be used.

Violence and violent activities can lead to increased farm abandonment and potentially reduced animal management activity, leaving stray cattle free to roam. In order to examine this effect, violent activity was collected using a media index for search terms related to border violence adjacent to the PQZ. An overall media index was calculated (*MediaAvg*) by averaging and re-indexing indices collected for the study region of various search terms related border violence, drug cartel violence, and Mexican drug cartel activities. These indices were gathered from Google Trends, a free online resource which captures the importance of a search or news topic for a given geography over time (25). In order to assess how well the Google Trends index reflected violent activity, we obtained a dataset from the Uppsala Conflict Data Program (26) that collects some records of violent activity (generally only activities that result in human deaths). A correlation of the media index with the available data series showed that *MediaAvg* was highly correlated (0.89) with the best death estimates available. The media trends provide an indication of the overall levels of violent activity, not just those that lead to deaths or hospitalizations, which serve as a better proxy for possible events influencing cattle abandonment. The media indices were collected from 2004 to 2019, which encompasses the full data available from Google.

To account for environmental factors that may lead to increase in stray cattle movements or changes in optimal tick habitation, weather data were collected from the National Oceanic and Atmospheric Administration's (NOAA) National Climatic Data Center (27). This data included hydrological and ambient data, such at maximum temperatures (*TempMax*) and precipitation indices (*PCP*). Drought indices can relate similar information through subtle calculation variations (28). For this analysis, the precipitation index most directly related to river levels in the Rio Grande River, a reduction of which may lead to easier crossing for livestock. The intention was to capture both the push and pull effects of environmental changes on cattle fever ticks and their hosts. Temperature extremes may explain livestock movements toward better grazing, while precipitation allows the effects of precipitation on river levels and forage growth to be modeled across the study region.

In order to capture the biogeographical CFT pressures, ecoregions were defined for the nine-county study region (Figure 1). The ecoregions identified in the PQZ were: Rio Grande Plains, Southern Gulf Prairies and Marshes, and Stockton Plateau as defined by Bailey (6). Where ecoregions overlapped in a county, the ecoregion that predominantly covered the county was recorded, leading to Southern Gulf ecoregion not being included despite covering part of Cameron County. While it would have been more accurate to record two ecoregions, this led to indicators representing individual counties rather than tick habitat suitability across counties. For the purposes of the analysis, Rio Grande Plains was considered the baseline and excluded to avoid multicollinearity.

**Figure 3:**
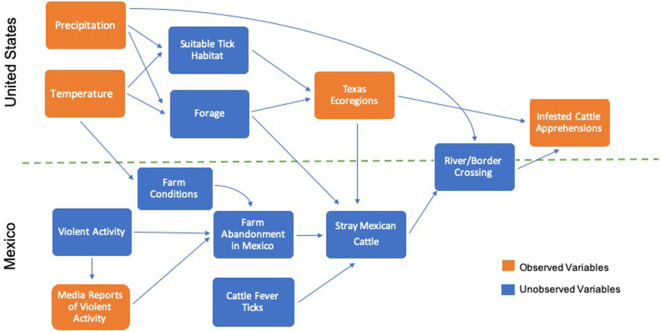
Conceptual model of directed relationships in Cattle Fever Tick analysis.

An overall trend variable (*Trend*) was included to capture temporal changes in annual cattle and tick movements not associated with the previous variables. A conceptual model is presented in Figure 3 which shows the directed relationship of the variables on infested cattle apprehensions.

### Methods

A panel modeling framework was used to determine the factors that contribute to CFT movements through infested cattle apprehensions. The panel nature of the by-county data over the study period provided a framework to estimate not only the effects across counties, but to best capture the temporal relationships between error terms. Due to the limiting nature of the media index and patrol hours, two sets of models were estimated. The first model group used the full set of explanatory variables and, empirically, the baseline model is represented in Equation (1):

(1) $$\begin{array}{r} InfestedBovine_{k,t}=\beta_{1}+\beta_{2}TempMax_{k,t}+\beta_{3}PCP_{k,t}\\ +\beta_{4}MediaAvg_{t}+\beta_{5}Trend_{t}+\beta_{6}RiverPatrolHours_{t}\\ +\gamma {\text{EcoRegion}}_{\text{k},\text{t}}+\varepsilon_{k,t} \end{array}$$

where β_*i*_ and γ_*i*_ represent parameters to be estimated, variables are as defined above for the kth county in time *t*, and ε represents the estimated error term. The first set of models (*All Effects Models)* are limited in study period from 2004 to 2019. In order to better understand the possible interactive effects of violence, tick habitat, and weather, a series of nuanced models were also estimated to examine first-order interactions for all predictors in the model.

In order to fully understand the longer-term effects of weather, a second set of models, *Long-Term Effects Models*, were estimated using the full 1978–2019 data. Similarly, the first order interaction models were also estimated. The baseline model to estimate the effects of weather on infestation counts over time is shown in Equation (2):

(2) $$\begin{array}{r} InfestedBovine_{k,t}=\;\beta_{1}+\beta_{2}TempMax_{k,t}+\beta_{3}PCP_{k,t}\\ \begin{array}{l} +\beta_{5}Trend_{t}+\gamma EcoRegion_{k,t}+\varepsilon_{k,t} \end{array} \end{array}$$

where all variables and parameters are the same as previously defined.

In estimating the empirical models, the panel Poisson estimator was used to better handle distinct, non-negative count data over the traditional ordinary least squares approach, which would treat *InfestedCattle* as a continuous variable, leading to less efficient results. The Poisson estimator is a pseudo maximum likelihood model with a log likelihood of Equation (2):

(3) $$\begin{array}{l} \text{log }L(\theta |X,Y)=\sum_{k=1}^{K}(y_{k,t}\theta x_{k,t}-e^{\theta^{\prime }x_{k,t}}-\text{ln}(y_{k,t}!)) \end{array}$$

where θ represents the set of parameters and *y*_*k,t*_ and *x*_*k,t*_ represent the observed counts and independent variables, respectively, for county *k* in year *t*. Optimal values of θ were determined through a search process of the pseudo-loglikelihood estimation.

One potential limitation of the Poisson estimator is that it makes an equidispersion assumption, which assumes the mean and variance are equal. If this assumption does not hold through overdispersion, a more generalized model, the negative binomial regression model, can be used. In order to test for overdispersion and to select the most appropriate model, a loglikelihood ratio test was estimated. Results failed to reject the hypothesis for equidispersion of mean and variance, thus confirming the appropriateness of the use of the Poisson estimator over a more generalized count estimator. Additionally, robust standard errors, clustering by county, were used for this analysis which helped to guard against underestimations of the error terms, a concern with Poisson estimators, as well as to address potential serial correlation in these terms.

## Results and Discussion

A summary of the CFT data at the county level is presented in Table 1 with a total of 286 county-level annual observations. Overall, there were 2,090 cattle apprehended over the time frame with an average of seven cattle (both non-infested and infested) apprehended in any given county per year, but this varies substantially with up to 70 non-infested and 64 infested cattle apprehended. As shown in Figure 1, the overlay of ecoregions on the counties in Texas shows much of the PQZ are centered in the Rio Grande Ecoregion. County-level infestation counts are presented in Table 2, note that Kinney county had no cattle apprehended, and Dimmit has limited observations with only one infested cow apprehended during the study periods due to their limited PQZ border area. As a result, these counties were excluded from the analysis, as previously discussed. There are counties that have a higher concentration of infested cattle apprehended, such as Val Verde, which on average has 10.5 infested cattle apprehended annually (max 64) compared to Zapata with 1 infested cow on average apprehended annually (max 9).

**Table 1:** Summary statistics for Cattle Fever Tick county-level annual data 1978–2019^a^^,^^b^.

**Variable**	**Units**	***N***	**Mean**	**Std. dev**.	**Min**	**Max**
Non-infested cattle	Count of cattle	286	3.7	7.4	0	70
Infested cattle	Count of cattle	286	3.6	7.8	0	64
Percent infested	Percent	286	36	41	0	100
River Patrol Hours^c^	Thousands of annual patrol hours for all counties	202	23.36	6.84	6.95	31.89
PCP	Precipitation index	286	1.95	0.45	0.93	3.05
Temp Max	Temperature (F)	286	83.85	1.53	81.18	87.28
Media average^d^	Index	104	44.75	23.91	10.75	100
Ecoregion: Rio Grande	1 if county in Ecoregion; 0 otherwise	286	0.72	0.34	0	1
Ecoregion: Stockton Plateau	1 if county in Ecoregion; 0 otherwise	286	0.28	0.45	0	1

a *Excluding Dimmit and Kinney counties*.

b *These summary statistics are derived from annual summaries for each county, as such the min and max represent the highest or lowest single annual county value, across all counties*.

c *Annual river patrol hours are limited to 1990–2019*.

d *Annual media index is limited to 2004–2019*.

**Table 2:** Annual county infested cattle apprehension summary 1979–2019.

**County**	***N***	**Mean**	**Std. dev**.	**Min**	**Max**
Cameron	41	2.1	3.5	0	18
Dimmit	12	0.2	0.4	0	1
Hidalgo	41	1.0	2.4	0	12
Kinney	13	0.0	0.0	0	0
Maverick	41	5.0	8.6	0	30
Starr	41	2.3	4.0	0	19
Val Verde	40	10.5	15.4	0	64
Webb	41	3.5	5.4	0	27
Zapata	41	1.0	2.0	0	9
Total^a^	286	3.4	7.7	0	64

a *Excluding Dimmit and Kinney*.

Selected modeling results are presented in Tables 3, 4. The incident rate ratios (IRR) represent the exponentiated coefficients and express the factor that the expected count of infested cattle apprehended change given a unit change in the independent variable. Both tables include baseline models, select first-order interaction models, and a combined, all interactions model, which presents interactions that are both theoretically sound and contribute to the understanding of the predictors influence on variability in infested cattle apprehensions.

**Table 3:** Incident rate ratio results for all effects models on Cattle Fever Tick infestation counts of apprehended cattle through the Cattle Fever Tick Eradication Program 2004–2019.

	**Baseline**	**Media interaction with ecoregion**	**PCP interaction with year**	**All interactions**
River Patrol Hours (thousands)	1.04^**^	1.05^***^	1.07^*^	1.07^*^
	(0.02)	(0.02)	(0.04)	(0.04)
Temp Max	1.18	1.15	1.32^***^	1.07
	(0.14)	(0.15)	(0.13)	(0.16)
Media average	1.01^*^	0.99	1.02^**^	0.66^*^
	(0.01)	(0.01)	(0.01)	(0.15)
Media average × Ecoregion: Stockton Plateau		1.03^***^		1.03^***^
		(0.01)		(0.00)
Media average × Temp Max				1.01^*^
				(0.00)
PCP × Year			1.32^**^	1.32
			(0.17)	(0.15)
PCP	1.23	1.12	0.00^**^	0.00^**^
	(0.95)	(0.89)	(0.00)	(0.00)
Year	1.13	1.15^***^	0.67^*^	0.67^*^
	(0.05)	(0.06)	(0.16)	(0.14)
Ecoregion: Stockton Plateau	5.11^***^	1.14	5.27^***^	1.00
	(0.01)	(0.70)	(1.73)	(0.67)
Constant	0.00^***^	0.00^***^	0.00^*^	0.00^***^
	(19.66)	(0.00)	(0.00)	(0.00)
Log Pseudolikelihood	−432.87	−413.19	−406.23	−380.19
*N*	104	104	104	104
Groups^2^	7	7	7	7
Mean *N* per Group	14.9	14.9	14.9	14.9

Results are incident rate ratios (IRR);

* p < 0.1;

** p < 0.05; and

*** *p < 0.01, Robust standard errors are in parentheses. Includes all quarantined counties except Dimmit and Kinney*.

**Table 4:** Incident rate ratio results long-term effects models on Cattle Fever Tick infestation counts of apprehended cattle through the Cattle Fever Tick Eradication Program 1978–2019.

	**Baseline**	**Region interaction with temp max**	**Region interaction with year**	**All interactions**
Temp Max	1.16^**^	0.99	1.16^**^	1.02
	(0.06)	(0.11)	(0.07)	(0.05)
PCP	0.96	0.95	0.94	0.94
	(0.32)	(0.30)	(0.30)	(0.10)
Year	1.03^**^	1.03^**^	1.01	1.02^***^
	(0.01)	(0.01)	(0.01)	(0.01)
Ecoregion: Stockton Plateau	4.08^***^	0.00^***^	0.00^**^	0.00^***^
	(0.34)	(0.00)	(0.00)	(0.00)
Ecoregion: Stockton Plateau × Temp Max		1.30^***^		1.20^***^
		(0.09)		(0.06)
Ecoregion: Stockton Plateau × Year			1.03^***^	1.02^***^
			(0.13)	(0.06)
Constant	0.00^***^	0.00^***^	0.00	0.00^***^
	(19.66)	(0.00)	0.00	(0.00)
Log Pseudolikelihood	−1,169	−1,149	−1,152	−1,145
*N*	286	286	286	286
Groups^2^	7	7	7	7
Mean *N* per group	40.9	40.9	40.9	40.9

Results are incident rate ratios (IRR);

* p < 0.1;

** p < 0.05; and

*** *p < 0.01, Robust standard errors are in parentheses. Includes all quarantined counties except Dimmit and Kinney*.

### All Effects Model Discussion

For the *All Effects Models*, which was limited to 2004–2019 due to data availability, river patrol hours, media-reported violence, and tick habitability were all significant predictors, and the results are discussed individually below. The *Temp Max, PCP*, and *Year* variables were not significant in the baseline model. These weather variables may not have been significant due to the relatively short timeframe covered in these models, which would impact the ability to measure how weather might drive changes in habitat suitability and tick/host migratory patterns. Despite this, temperature did show an effect on apprehensions when related to media-reported violence. In the All Interactions model, the interaction between *Media Average* and *Temp Max* is estimated to affect infested cattle apprehensions by a factor of 1.01, which implies that the effects of media-reported violence differ given the temperature. In the *Long-Term Effects Model*s presented later, the change in temperature and an overall trend shows a significant relationship across all models. The exact reason is unknown but these results may indicate a relationship between temperature and ranching conditions that may induce livestock to stray, such as drought conditions (e.g., low rainfall and high temperatures) or shorter-term shortages in forage, hay, or water.

Patrol Hours (*River Patrol Hours*) was a significant predictor in all models. While the average number of patrol hours in the dataset was 23,000 h/year, for every additional 1,000 h of patrol annually, the likelihood of additional counts of infested cattle increases by 4–7% (Table 3). This implies that the number of hours patrolled has a significant effect on capturing infested cattle. In estimating all first-order interaction, the effect of patrol hours varied significantly (*p* < 0.01) with weather variables (*Temp Max* or *PCP*) or with *Year* (not presented in Table 3); however, both interactions could not be combined into a single model due to collinearity issues. However, these results suggest that the relationship between river patrol hours and infested cattle apprehensions may be impacted by climate variability. The relationship between climate variability and river patrol hours also increases the impact of the river patrol hours meaning that during periods of increased climate variability an increase in river patrol hours can compensate for this volatility.

Thinking about these results in another way, model results show that reducing the number of patrol hours would directly reduce the number of infested cattle apprehended, which may lead to reinfestations, high (nearly 90%) mortality rates, and high control costs (5). Recognizing the value of tick rider patrol hours is useful in understanding the effectiveness and importance of the horseback patrols and the financial support of vigilant surveillance in the PQZ.

Like river patrol hours, the media-reported violence indices showed a significant effect (*p* < 0.1 and *p* < 0.05) in the baseline and interacted models. During times of increased media reporting of violence and violent activities in the PQZ, the likelihood of infested cattle apprehensions increases. For example, using the baseline model, an increase by one point in the media index increases infested apprehensions by 1%. For the *All Interactions* model, media-reported violence appears to vary based on location (ecoregion) and temperature. While the mechanisms for these effects are not clear based on the available data, it is possible that periods of intense violence and violent activities could lead to an increase in farm or cattle abandonment leading to increased stray animals. The significant effect of media-reported violence and violent activities on farm abandonment reinforces the literature in the broad effects of social and political conflicts outlined by Maldonado Aranda (23) and Deraga (24). In addition to farm abandonment, cattle may be triggered to move due to violent or loud, disruptive activity in their home ranges. The relationship between media-reported violence and infected cattle apprehensions highlights the need for further sociologic work in this region, which may provide a more complete understanding of the driving forces behind CFT pressures in the PQZ.

The largest influencing factor on CFT apprehensions was tick habitability. There are significant (*p* < 0.01) differences in ecoregion-estimated apprehension counts across the baseline and interacted models for the Rio Grande and Stockton Plateau ecoregions. Stockton Plateau ecoregion is expected to have 5.11 times greater rate of infested cattle apprehensions than Rio Grande ecoregion in the baseline model. Additionally, the effect of ecoregion appears to vary based on media-reported violence, such that there is a significant (*p* < 0.01) difference in the effects, a factor of 1.03 for the Stockton Plateau over the Rio Grande ecoregion, of media reported violence and violent activity by ecoregions. This variability could indicate either heterogeneity in the location of violent activities or that the movement patterns of stray animals have some preference during times of high stress, such as abandonment. The difference in apprehension rates is interesting, since the ecoregions vary in terms of accessibility, visibility, and desirability of hosts moving across the border. A combined understanding of where infested cattle are more likely to enter into the PQZ and where tick habitat and host availability is most likely to allow establishment of the tick could be used to prioritize labor or resources during emergencies, such as outbreaks of other diseases, that may draw resources to other areas.

### Long-Term Effects Model Discussion

The *All Effects Models* provide an understanding of a broader set of explanatory factors that may contribute to tick infestations and apprehensions, but they are limited to a smaller sample of data. In order to understand the long-term effects of weather changes on infested cattle apprehensions, the *Long-Term Effects Model* was estimated. Directionally, the results are consistent with the previous model; however, this model has the benefit of estimating the overarching effects of the climate as well as temporal and spatial effects. Results are shown in Table 4.

In terms of temperature, the *Long-Term Effects Models* shows a significant effect (*p* < 0.05–0.01), such that for every one degree increase in the average maximum temperature by county, the number of cattle apprehended is 16% greater than the expected counts. This change in apprehension shows that over the full data series, weather could be driving changing patterns of livestock movement or changes in tick habitat suitability. These results provide additional indication of the effects of changing weather patterns on tick habitats that was discussed in the literature by Estrada-Peña (20). Additionally, the effects of weather are not uniform across all regions. When accounting for the effects of temperature across the entire time frame, within the Stockton Plateau ecoregion, there is a 1.30–1.20 times greater rate in the number of infested cattle apprehended compared to the Rio Grande ecoregion (*p* < 0.01). These regional, climactic effects suggest that weather changes may affect tick habitat suitability and these changes are unlikely to be homogenous across the region. This may require ecoregion-specific approaches to dealing with weather patterns deviations within the PQZ.

The ecoregion-estimated IRRs for the *Long-Term Models*, while consistent with the results of the *All Effects Models*, are slightly more conservative. In the baseline model, Stockton Plateau ecoregion had an infested cattle apprehension rate 4.08 times higher, compared to the Rio Grande ecoregion. Building on the previous discussion, the effect of ecoregion also varies by year. Ecoregion-year interactions indicate a 1.02–1.03 increase in infested cattle apprehensions for Stockton Plateau for each an additional year. This slope adjustment implies that in addition to weather changes varying by region, temporal changes also vary by region, which speaks to the heterogeneity of counties within the PQZ. An explanation for these temporal effects could be heterogenous land use and management changes over time within ecoregions. Overall, these results reassert the importance of understanding how and where infested cattle introductions change over time, as well as understanding the complex interactions of weather and ecoregion-specific factors on the success of apprehensions.

Following the temporal changes by ecoregion, there was a significant estimated annual change in infested cattle apprehensions over the study period. For each additional year, counts of infested cattle apprehended is estimated to increase by 2–3% across all models. The long-term increase in infested cattle movements over time is consistent with continued pest pressures, reaffirming the value of the PQZ activities in monitoring for reincursions. These estimated effects are more conservative than in the *All Effects Models* (13% increase), which may indicate changes in PQZ management practices over time or may show an increased pressure of infested cattle during the previous 15 years vs. the entire 41-year period examined in the long-term effects model. Further research into tick management in endemic areas and habitats may shed more light on the causative factors associated with these increases.

## Conclusions

Ectoparasites, such as cattle fever ticks, and the diseases they carry pose a risk to the global cattle population in reduced productivity and in livability. Cattle fever, bovine babesiosis, was once endemic in US cattle, but the disease was eradicated through concerted and costly efforts across agencies and producer groups to eradicate the cattle fever tick. Reintroduction of this disease to the US cattle herd could lead to substantial mortality and costs in terms of containment, eradication, and effects on producers and consumers. A permanent quarantine area provides constant surveillance for reincursions from endemic areas to minimize those risks. However, these risks vary due to a variety of factors, leading to fluctuating pressures on tick and host migrations into the permanent quarantine area.

The number of infested cattle apprehended in the permanent quarantine area has increased over the last several decades. By analyzing factors that help explain the variability in the number of infested cattle apprehended, this analysis provides a better understanding of how pressures for tick reintroduction, in the form of infested cattle, have and continue to change. The results from this analysis suggest that both media-reported violence and weather changes are associated with the rate at which infested cattle are apprehended, and these effects differ depending on spatial and temporal factors. With continued land use changes, social unrest in endemic areas, and changing weather patterns, the efforts to control and eradicate CFT, both in the United States and globally, is likely to be an ongoing concern. Control efforts which take into account these factors in addition to host/parasite ecology may be more successful in long-term prevention of reestablishment of the cattle fever tick in the U.S.

Continued study on the value of apprehending infested cattle and the mitigation of the risks and costs associated with reincursions is vital. Additionally, an economic analysis on the value of maintaining a permanent quarantine could create a deeper understanding of CFT control programs and impacts. For the U.S. to continue to be successful in controlling CFT, multidisciplinary and targeted approaches will be needed to account for changing CFT pressures as well as new and evolving control measures.

## Data Availability Statement

The datasets generated for this study are available upon request but may be subject to USDA approvals. Requests to access these datasets should be directed to Jada Thompson, jthom207@UTK.edu and Hallie Hasel, hallie.s.hasel@usda.gov.

## Ethics Statement

The manuscript data used are secondary data with no identifying information for humans or animals. We used surveillance data provided by USDA:APHIS and other publicly available datasets to run the statistical models. The surveillance data is collected as part of the ongoing USDA Cattle Fever Tick Eradication Program. As such there are no ethics review needed for this paper.

## Author Contributions

JT was the project leader, estimated the statistical models, and lead manuscript development. AD led effects modification analysis and contributed to manuscript development. HH compiled data, provided expert advice, and contributed to manuscript development. DB coordinated group communication and contributed to manuscript development. All the authors met the contribution requirements for authorship per the author guidelines.

## Conflict of Interest

The authors declare that the research was conducted in the absence of any commercial or financial relationships that could be construed as a potential conflict of interest.

## Footnotes

^1^About the Rio Grande Ibwc.gov. Available online at: https://www.ibwc.gov/CRP/riogrande.htm (accessed May 26, 2020).

^2^The year 2014 is excluded from the analysis as it represents outlier period where ~200 cattle were requested to be moved by the CFTEP. However, these cattle had lived in the northern part of the PQZ (an area not environmentally optimal for tick development) for more than a decade and does not represent cattle apprehended and checked for cattle fever ticks in the course of normal operations.
